# Black Women Faculty and Administrators Navigating COVID-19, Social Unrest, and Academia: Challenges and Strategies

**DOI:** 10.3390/ijerph19042220

**Published:** 2022-02-16

**Authors:** Anuli Njoku, Marian Evans

**Affiliations:** Department of Public Health, College of Health and Human Services, Southern Connecticut State University, 144 Farnham Avenue, New Haven, CT 06515, USA; evansm7@southernct.edu

**Keywords:** COVID-19/coronavirus, race/ethnicity, health disparities, racism, diversity, Blacks/African Americans, Black women faculty, Black women administrators, higher education, equity

## Abstract

Black women faculty and administrators in the United States are tackling a force of socioeconomic and racial disparities, emotional tolls and invisible burdens within academia, political turmoil, social unrest, and public health crises. COVID-19 has added an additional layer related to work responsibilities, the overall well-being of Black women faculty and administrators and the diverse students they encounter, and management of work and home responsibilities. This paper discusses perspectives and evidence-based strategies regarding Black women faculty and administrators who navigate academia and teach during times of COVID-19 and social unrest. We also outline strategies for university leaders to mitigate cultural and racial gaps in the classroom or workplace and foster diversity and inclusion in academia.

## 1. Introduction

The year 2020 was tumultuous in the United States (U.S.); it included the beginning of a global pandemic, economic fallout for people who were already economically vulnerable, and a rise in populism. Moreover, the murder of George Floyd alongside higher COVID-19 health disparities for Black and Hispanic (or Latinx) communities revealed centuries of socioeconomic disparities as well as racial and ethnic discrimination [1]. The Civil Unrest Index predicts that these intersections will serve as a catalyst for future unrest in the U.S. In 2021, there were unprecedented sieges and deaths, along with the insurrection of Capitol Hill incited by the highest level of leadership in the country. This level of civil unrest has not been seen since the Civil Rights Era. The COVID-19 pandemic has exacerbated and revealed the fragility of mental health and measures of instability in the U.S. [2,3,4]. This is due to the indirect impact of infectious disease measures instituted to contain and control the spread of COVID-19 on national and global levels. The COVID-19 pandemic has keenly reinforced the stark truth that, in the U.S., Black, Indigenous, and People of Color (BIPOC) communities not only suffer but die in larger proportion than Whites [4,5,6,7,8,9]. Racial and ethnic disparities in suffering and deaths are manifesting through COVID-19 as well as through the outright brutalizing and killing of Black persons at the hands of law enforcement [10]. In higher education, the rapid pivot response to COVID-19 with remote and virtual work and learning has taken a dramatic toll on college students’ mental health and highlighted multiple levels of mental health trauma and disparities. For example, Black students show elevated levels of depression when compared to White students [11]. COVID-19 and its revelations have also compounded the endemic trauma that normally exists for BIPOC staff, faculty, and students [12].

Furthermore, COVID-19 has also exacerbated inequities among faculty of color, especially Black women faculty, as a microcosm of the larger social context [13]. Black women, particularly, are encountering physical, mental, and financial challenges during this unprecedented time [14,15]. As COVID-19 takes disproportionate health and financial tolls on racial and ethnic minority groups in the U.S., pre-existing inequities among faculty members are amplified. Faculty of color, particularly Black faculty, and Black women, are more likely to be coping with family illness, homeschooling of children, unemployment, loss of loved ones, and caregiving while also providing emotional support to struggling students [16]. This encourages the exploration into how Black women faculty and administrators can teach and work in higher education while dealing with this trauma and civil unrest, identify resources for support, and continue trauma-informed practices with their students. Moreover, there are action steps that academic institutions can take to support Black women faculty and administrators who navigate these issues. This article describes Black women faculty and administrators’ perspectives and strategies during times of COVID-19 and social unrest. We detail cultural and racial experiences in academia to bridge gaps and foster diversity and inclusion in classrooms and workplace environments. We also outline effective strategies for university leaders to mitigate racial inequities for Black women in academia.

## 2. COVID-19 Disparities as a Public Health Crisis

SARS-CoV-2 is part of a large family of coronaviruses that cause respiratory diseases. This viral strain was first detected in 2019 and designated as COVID-19, a virus of concern (VOC) that became a global public health pandemic in March 2020. Several variants are tracked globally but have not yet risen to the status for concern. There are two dominant variants (Delta and Omicron) that have emerged as a VOC due to their potential to spread rapidly and cause severe morbidity and mortality in unvaccinated persons. To date, over 947,000 people (about half the population of the U.S. state of Idaho) in the U.S. have died, over 79 million people have been infected, and over 547 million people have been vaccinated [17]. While everyone is at risk of infection, some people with chronic conditions and the unvaccinated are at a higher risk of morbidity and death. Racial and ethnic minorities have carried the brunt of disease burden and mortality in the U.S. for the most acute and chronic conditions, as seen in the data [5]. COVID-19 infections have doubled and tripled the historical suffering in racial and ethnic minority communities in the U.S. due to pervasive structural barriers and racism [18]. The current public health, racial, and economic crises brought on by the COVID-19 pandemic has had a disproportionately negative impact on historically underserved individuals and communities of color. While disparities have existed in the U.S. for centuries, COVID-19 inequities have further illuminated the lingering gaps in health outcomes between communities of color and their White counterparts. These inequities are not new, but COVID-19 has further revealed them. Overall, COVID-19 disparities are one of the intersecting afflictions affecting Black Americans during this pandemic.

The COVID-19 pandemic has been the tipping point for showcasing the fragility of the country’s infrastructure and inequitable distribution of resources for decades. The public health field has improved at tracking, counting, and monitoring (surveillance) but not at undoing or eliminating disparities due to the root cause of racism. People with lower socioeconomic status in the U.S. suffer more death, higher morbidity, hospitalizations, and mortality in most chronic conditions as well as COVID-19 [18]. Because Black Americans are twice as likely as Whites to know someone who has been hospitalized with or has died from COVID-19 [19], this can also produce a second-hand trauma that exists when they repeatedly witness the pain and suffering of people who look like them [20]. Not until we develop equity-focused public health solutions that undo the effects of systemic racism will these disparities dissipate for people of color. Within academia, the COVID-19 pandemic has produced gendered, racial, parental, health, and other impacts to work-life balance and academic productivity, with Black women being unfavorably affected [13,21]. These factors warrant attention to the role of social determinants of health, stigma, and trauma produced during the COVID-19 pandemic and how Black women in academia can be adversely affected.

## 3. Police Brutality, Social Unrest, and Political Turmoil

Black Americans have been disproportionately impacted by police brutality, both before and during the COVID-19 pandemic [18,22,23]. Police brutality has been brought to the forefront as a systematic injustice that Black Americans face due to the horrific killing of George Floyd on Memorial Day, 25 May 2020, (as well as those of other Black people within that period), and the ensuing international anti-racism protests against police brutality [10]. Research has shown that the various means through which police brutality may increase the death rates among the Black community include overrepresentation as victims of physical injuries and deaths, racist public reactions, psychological stress, economic and financial strain, and systematic disempowerment among the Black community [22,24]. Racial and health disparities worsen in the face of public health emergencies such as disasters and pandemics [25,26]. Furthermore, police brutality may increase the transmission of COVID-19 among the Black community due to chronic stress caused by persistent police-related trauma, police presence, incarceration, and potentially deadly encounters [27,28,29].

Instances of police brutality can affect Black women in the academic work environment. The year 2020 created increased emotional trauma for Black employees and a work from home lifestyle during a pandemic. Black women often felt relieved to work from home, free from microaggressions and away from workplaces with co-workers who may not have sympathized with this emotional trauma [30]. A national survey among U.S. workers has shown that Black women have a unique experience in the workplace and are the least likely to feel valued and treated with respect compared to other groups [31]. Moreover, the students that Black women in academia teach and serve are also affected by police brutality, as research shows that publicizing police killings of unarmed Black people causes emotional trauma (for example, anxiety and fear) among Black and Latinx college students [32]. Such racism fuels poor mental health outcomes among Black students and hurts grades and graduation rates [32], which is an added consideration for Black women who teach, mentor, supervise, and serve these students.

Seeing increased media coverage of police brutality can create a vicarious trauma that exists when Black Americans repeatedly witness the pain and suffering of people who look like them or remind them of their loved ones. Moreover, the Black community, including workplace employees, can be traumatized by viewing graphic images or videos of police brutality [33,34]. Furthermore, police brutality is a psychosocial stressor that can play a huge role in affecting Black mothers’ lives. Black mothers experience a gendered racial vulnerability by often being responsible for teaching their children how to respond to police violence in the “police talk” [35], which can cause psychological distress and physical manifestations of stress [36]. Police brutality and other forms of structural racism can also take a toll on Black mothers, including those in the workplace, as anticipated negative police-youth encounters have been linked to depressive symptoms among pregnant Black women [37,38]. Furthermore, Black women are not exempt from racially toxic classrooms and gendered violence within higher education [39].

Black women in academia have also had to work amid racially divisive, violent national uprisings. On 6 January 2021, a vehement attack occurred at the United States (U.S.) Capitol in Washington, D.C., during which rioters attempted to overturn the results of the 2020 presidential election [40]. Many noticed the clear racial double standards during which there were merely a few dozen arrests among the Capitol insurrection group of overwhelmingly White Americans, in contrast to the overwhelming force from law enforcement and more than 14,000 arrests during the 2020 Black Lives Matter protests [41]. College and university presidents, provosts, and higher education leaders across the country strongly condemned the violence at the U.S. Capitol, with the evolvement of longer and more formal statements in comparison to initial reactions [42]. With COVID-19, police brutality, anti-Black violence, and revolts such as the insurrection at the U.S. Capitol causing further erosion of trust in institutions and schools and by Black Americans [43], Black women still had to return to work to lead, teach, and serve, often without the privacy to grieve mounting racial unrest [31]. Teaching in the classroom after the insurrection has also resurfaced questions regarding who belongs in the classroom, who is othered, and how to manage conversations about the nation’s unresolved legacies of systemic injustices, magnified by the display of rioters at the Capitol [44]. Increased racial trauma and mental health concerns will have salient implications for teaching and learning post-pandemic [45]. Furthermore, Black women in the workplace will be adversely impacted by economic inequality in the COVID-19 labor market [46].

## 4. Black Women in Higher Education

With the occurrences of COVID-19 disparities, lingering economic equities, and social unrest, continued insight is needed into Black women’s experiences as faculty and leaders in higher education [47]. Black faculty and other faculty of color have been continually underrepresented in higher education [13]. Of all full-time faculty in U.S. degree-granting postsecondary institutions in fall 2018, approximately 40 percent were White males; 35 percent were White females; seven percent were Asian/Pacific Islander males; five percent were Asian/Pacific Islander females; three percent each were Black males, Black females, Hispanic males, and Hispanic females; and American Indian/Alaska Native persons and those who were of two or more races, each made up one percent or less of full-time faculty [48]. Even though the percentage of all doctoral degree recipients who are Black has gradually increased each year, Black faculty still comprise less than six percent of full-time faculty at U.S. colleges and universities [49]. Although Black female students earn 67% of all doctoral degrees granted to Black students [50], the percentage of Black women who hold associate and full professor positions in higher education has not increased considerably [48]. Furthermore, Black women are underrepresented as full-time faculty members in higher education, particularly in tenure-track positions [13]. Overall, women of color are disproportionately underrepresented in higher education administration, particularly at the presidency level, making up only 5% of all college presidents [51]. Altogether, these racial inequities show there is still much room for improvement.

### 4.1. Workplace Discrimination

Workplace discrimination is prevalent in the experiences of Black women working as faculty in higher education [52]. Research in this area uncovers the occurrence of negative stereotypes steeped in racist, sexist, and classist beliefs from the era of slavery about Black women as unworthy recipients of affirmative action [53] and as individuals who lack workplace productivity [54]. These stereotypes lead to fewer employment and educational opportunities and lower wages for Black women faculty in higher education compared to their White male counterparts [55,56]. This intersecting level of racial and gender discrimination persists at every level of academic life, leading to faculty and students viewing Black women scholars as less capable and resulting in fewer full-time, tenured positions for Black women faculty [52]. Addressing these issues is further complicated as Black women seldom hold administrative positions in universities [52] and are underrepresented in higher education leadership [47]. The history of Black women in U.S. higher education can be described as a lesson in persistence, courage, and overcoming diversity, complete with persistent challenges, such as the gender pay gap, the concrete ceiling, regulated availability, power struggles, limited role models, privilege, tokenism, invisibility, microaggressions, and isolation [57,58]. Furthermore, many Black women who occupy influential positions such as tenured faculty and administrative leaders at predominantly White institutions exhibit a degree of fear and caution with their hair expression or adjust their hair to fulfill professional norms in spaces where they have already substantiated themselves as qualified [13,59]. Black women face a multilayered oppression due to European cultural influences on Black American ideas regarding beauty, hair, and identity and what is deemed as acceptable hair expression in the workplace environment [59]. There is a need to consider the lived experiences of Black women who navigate hair expression while working in predominantly White institutions [13].

Overall, the intersections of race and gender overpower Black women to a fundamentally different reality than White women in the U.S. and result in the experience of several forms of oppression, including, but not limited to, racism, sexism, and classism [60,61]. Moreover, navigating the intersection of both race and gender can be challenging for Black women in academia, particularly when they lack proper support, mentorship, and representation; must pursue and persist in academic careers; see the effects of race and gender on their leadership development and career trajectories [62]; and must balance expectations with authenticity [63].

### 4.2. Implications for Black Women in Academia during COVID-19

Students, faculty, and administrators alike have all scrambled to pivot and persevere during the COVID-19 pandemic. It has also aggravated inequities among faculty of color, especially Black women faculty, when as a microcosm of the greater societal context. Experiences of racial fatigue, frustration, alienation, isolation, exhaustion, overextension, and undervaluation are common sentiments shared by Black women faculty, having a negative impact on their mental health, academic scholarship, and career trajectories [13,16]. With women more likely to participate in caregiving full-time and men to produce scholarship [64], there has been gender inequities in research productivity during the COVID-19 pandemic [65]. These inequalities in scholarship have been exacerbated for BIPOC women during the pandemic [15].

The COVID-19 pandemic has contributed to the magnification of gendered roles at home, and the lack of division of shared household tasks and work. Overlapping roles of teacher, babysitter, wife, sister, caregiver, and all-day cook/chef reveal the amplification of roles and expectations for Black women faculty and the intersection of their responsibilities at work and home. During the COVID-19 pandemic, Black women faculty are more likely to tackle personal struggles (for example, family illness, loss of loved ones, unemployment, caregiving, and home schooling of children) while attempting to provide support to struggling students [16]. Systemic inequity with the intersection of race, gender, and class increases marginalization and financial hardships for Black women faculty. Qualified academics are often caught in academia’s continuous underclass, with research showing that colleges hire more minority and female professors for adjunct jobs rather than tenure track positions [66,67]. Education experts state that pandemic-related layoffs and hiring freezes illustrate that faculty from underrepresented backgrounds will be among the first to be fired [68]. Regardless of tenure status, this is far from comforting news for academics. Data also shows that colleges and universities not being deliberate about increasing diversity in academia will only heighten the inequality emphasized by COVID-19 [68].

### 4.3. Black Women Administrators

Black women have always been leaders in education. For the past two centuries, during the civil rights years and throughout, student protests and demands at predominantly White institutions (PWIs) have repeatedly called to increase the number of Black faculty and administrators [69,70]. There is a scarcity of data published with the unique locale of PWIs and Black women administrator perspectives due to the small number of Black women that exist and remain in these positions of leadership. In the U.S., less than 10% of leaders at PWIs are Black, with an even smaller proportion of this group being Black women administrators. [71,72]. More insight is needed into the perspectives and sentiments of Black women administrators during the times of COVID-19 and civil unrest. Black women administrators are dealing with compounded personal struggles and sentiments similar to faculty, as mentioned above, along with compounded professional challenges (e.g., student retention, quarantine, programming, decreased enrollment, graduation rates, resource gaps, and campus COVID-19 infection rates) while attempting to provide all hazard support to struggling and traumatized students and faculty [16,73,74].

During 2021, the authors at various times held informal but timely conversations with several Black women administrators across the country, who self-identified as Black administrators at PWIs. These discussions occurred during presentations at academic conferences, informal virtual dialogues, and ongoing gatherings. Discussants varied in age and experience as well as educational backgrounds. They were at various levels in academia and worked at both public and private four-year institutions. These discussions brought important voices to the forefront, highlighting the potential long-term impacts of COVID-19 in academia, and providing insights into how leadership can intervene to address barriers faced by Black women administrators [72]. Overarching themes arose, such as the resounding need for strategies for self-care in all forms. Other concerns included racial fatigue, racialized motherhood and pregnancy challenges, remote work challenges, teaching from home coupled with and without homeschooling, hair management and expression anxiety, camera and Zoom fatigue, the trauma of violence (for example, the murders of George Floyd, Breonna Taylor, Ahmaud Arbery, and Michael Brown), repetitive traumatization due to increased media coverage of police brutality, difficulty with caregiving tasks, the emotional and physical toll of grief and loss, and the overall challenges of managing expanded roles and expectations. All of this proved what Nicol and Yee explained as follows: “We know that we cannot sustain ourselves if we are physically exhausted, emotionally drained, and spiritually dead” [75] (p. 135).

## 5. Discussion

Black women administrators and faculty continue to work and teach through trauma and civil unrest at a predominantly White institution. COVID-19 and its revelations has compounded the endemic trauma of staff, faculty, and students that normally exists. How we support each other as Black women administrators and faculty and continue trauma-informed practices with our students is crucial.

Strategies are needed to effectively address the challenges faced by Black women in higher education who are also grappling with a global COVID-19 pandemic and racial and social unrest [13,14]. Below are individual and institutional-level strategies for Black women to navigate academia during COVID-19 and unrest.

### 5.1. Individual-Level Strategies

#### 5.1.1. Practice Self-Care

Self-care is a commonly used word that encompasses multiple meanings. Overall health disparities have persisted among Black women before and during this pandemic [65]. This implores the need to stay current on screenings for chronic conditions such as cancers, hypertension, diabetes, and other ailments to promote health and prevent disease. Other strategies to promote health and self-care include sharing thoughts, exercising, practicing yoga, obtaining massages and facials, seeking therapy and professional help to feel less isolated [13], obtaining adequate rest and sleep [13], and accepting help with both work and caregiving tasks [68]. Another recommendation is to explore available campus resources such as mental health support and counseling programs. It is also important to consider the “3 G’s”—Go with the flow, Grace, and Gratitude. As Black women toil and persevere within academia during these times, it is important that they give themselves grace. This includes acknowledging resilience in the face of adversity and giving oneself permission to rest [13]. Giving latitude includes looking at one’s teaching, scholarship, and service load and then determining if their work aligns with their research and career goals and if there are tasks that can be delayed or not done [69]. Another approach is to consider home life and adjust expectations accordingly [68]. Other strategies include practicing mindfulness and embracing an attitude of gratitude. Writing in a journal can be beneficial to help relieve stress, improve writing, set, and achieve goals, boost memory, and inspire creativity [70].

#### 5.1.2. Block Out Time

Another self-care strategy is to intentionally block out time on one’s work calendar (e.g., Fridays; days in the week of a particular holiday). Administrators can make use of their personal assistant (literally or figuratively). If an individual does not have a personal assistant, they can be their own. If there are pockets of time that spur creativity, they should block these out on their calendar. Given the gender and other disparities in scholarship observed during the pandemic [15], blocking out time for writing time may encourage productivity [68]. Carved out time can also be used to plan family trips and excursions. Planning ahead for trips may also help save money on costs compared to a last-minute booking.

#### 5.1.3. Connect with Your Circle

Humans are interpersonal beings, and it is important to connect with one’s circle during these times. This can include leisure activities such as taking a campus walk with co-workers and meeting peers for lunch, even if virtually. For Black women in academia, supportive communication with friendship networks and check-ins with peers can help provide socio-political contextualization, collective upliftment, and enjoyment [71]. Networking among women in academia can be organic and based on shared interests with peers, or it can be strategic and focused on career trajectories [72].

#### 5.1.4. Professional Development

As discussed, COVID-19 has hindered productivity for academic women, with Black women adversely affected [15]. For those on the tenure track, it has meant publishing less due to having extra home and work responsibilities [13]. With institutional support, academic women can gain professional development by attending trainings and webinars and keeping their membership and certifications current. Professional development can provide continuing education and keep individuals marketable and current on the latest research and best practices. Professional development can also be in the form of conference attendance, both online and in-person, to learn and share ideas, inspire creativity, expand one’s network, and build one’s portfolio. In-person conference attendance can allow for rejuvenation, a change of scenery, and in-person socialization.

Recognizing that institutional involvement is also needed to mitigate socioeconomic and racial inequities for Black women in academia, we propose the following recommendations as starting points for institutional leaders (Table 1):

### 5.2. Institutional-Level Strategies

#### 5.2.1. Genuinely Commit to Diversity, Equity, and Inclusion

Institutions that authentically commit to diversity, equity, and inclusion can foster candid conversations about privilege, power, and oppression [14]. There must be recruitment and retention of faculty and staff with diverse experiences and perspectives. Representation matters, and there is an ever-present need to recruit and retain BIPOC faculty. Academic institutions must actively recruit and retain racially diverse faculty who have diverse experiences with racism; ensure cultural competence and mentoring knowledge among their current faculty; and be dedicated to recruiting, retaining, and graduating students from diverse backgrounds [73]. Institutions should directly acknowledge and address the “minority tax” and avoid overburdening faculty from historically underrepresented groups [69]. There should be a call to allies to intentionally learn how anti-Black racism functions in academia and to teach about these concepts [74]. There should also be institutional commitment to provide and sustain racial awareness training for faculty. Such trainings may positively influence the ability and motivation of faculty to implement strategies to promote student learning regarding racism and its impact on health and the promotion of social justice. Continued racial awareness, diversity training, and a commitment to anti-racism for faculty and practitioners are critically important due to our increasingly diverse, yet divisive, society [77]. Furthermore, such trainings have been shown to improve classroom dynamics, teaching, and assessment; have a positive effect on instructors’ and students’ personal growth; and result in attitudinal and curricular changes [76,77].

#### 5.2.2. Change Policies to Offer Flexibility

There have been calls from professional organizations and scholarly societies to adjust expectations for faculty scholarship during this period [15,16]. Recommendations include pausing nonessential scholarships, limiting meetings, waiving nonessential service, delaying the tenure clock, and shifting institutional norms [14]. Institutions can promote flexibility by changing teaching policies to benefit faculty. For instance, institutional leaders can recognize the influence of gender and racial biases in student evaluations and incorporate holistic teaching assessments [16]. There can also be a limit in the use of student evaluations of teaching from the recent term for both tenure-track and provisional faculty [15]. Institutions should also offer more telework options to help employees effectively manage home and work responsibilities [13].

#### 5.2.3. Fund Professional Development

As COVID-19 has impacted faculty members’ careers, disrupted research activities, and led to gender inequities in scholarship [15], institutions can fund professional development to support employees during COVID-19. If pause in faculty travel for conferences during COVID-19 created a surplus of funds, institutions can roll over unused travel funds to support future faculty travel. Furthermore, such funds can support professional development for faculty and staff, including sponsorship to travel to academic conferences and maintain professional memberships.

#### 5.2.4. Recognize Intensified Caregiving Demands

Institutions must recognize that the pandemic has changed the personal and family lives of faculty members, taken substantial health and financial tolls, and adversely impacted their work. One area of note is the increased caregiving demands, particularly among women faculty members, and the unfavorable impact on their work [64]. Institutions can respond to this need by providing emergency funds for caregiving assistance, including childcare and eldercare [16]. Other strategies include promoting the availability of counseling and mental health services, incorporating a day of no classes, and organizing campus events to relieve stress and build morale. Moreover, providing increased academic and social emotional support for students will ultimately help the workload of administrators and faculty.

#### 5.2.5. Trauma-Informed Practices

Trauma is an emotional response to a terrible event, such as an accident, rape, or natural disaster. Immediately after the event, shock and denial are typical emotional responses. Longer term reactions include unpredictable emotions, flashbacks, strained relationships, and even physical symptoms such as headaches or nausea [79,80]. Adverse childhood experiences (ACEs) are potentially traumatic events that occur in childhood (0–17 years), and include experiencing or witnessing violence, the death of loved ones, and mental health and substance abuse problems in the home. These events can all have chronic physical health effects and are linked to engagement and behaviors throughout the lifespan [79,80]. It is important to think about a trauma-sensitive informed approach to pedagogy for students as well as adults, especially during this time of unrest and chaos, which is traumatizing in real-time. ACEs and trauma go hand in hand, and it is important that more people in higher education recognize and adapt trauma-informed practices inside and outside of the classroom and in the workplace to create a culture of health and resiliency in a campus community [79]. Trauma-informed pedagogy practices can be as simple as checking in with students, faculty, or staff; being flexible when possible; valuing engagement of all types; and creating a sense of belonging throughout the institution. In a study carried out by Carello and Butler [81], researchers found that many students attending college have already experienced trauma and post-traumatic stress disorder (PTSD). Experiencing trauma affects the overall health and wellbeing of all. Therefore, a trauma-informed approach to everyday work, service, collaboration, communication, and pedagogy needs to be implemented and recognized as being of the utmost importance during this pandemic [81,82].

#### 5.2.6. Foster Collaboration and Communication

The rapid pivoting during COVID-19 presented numerous challenges in teaching, classroom learning, and work practices. Engagement has been one of the most challenging aspects of teaching and remote learning during COVID-19 [83]. One of the lessons learned during COVID-19 is that outdoor community service learning and group work are opportunities paramount to student engagement. Technology (e.g., Zoom, Webex, Teams software) brought the convenience of rapid collaboration and communication, the ability to make external connections, and the opportunity to build positive role-playing experiences virtually. Role-playing allows the building and practice of social and emotional skills with peers in small group settings (e.g., breakout rooms). Studies show that fostering collaboration and communication builds skills to engage in positive discourse, improves achievement and satisfaction, increases understanding of roles and expectations, lowers barriers to communication, increases value and respect for others, creates safe learning environments, and improves the ability to probe and ask questions that reveal a deeper acquisition of critical thinking skills [78,83]. There is also a need to allow for open dialogue between faculty members and administrators. Institutions must create safe and welcoming spaces—such as virtual town halls—where faculty members can express to administrators their concerns regarding COVID-19′s effect on their careers. Administrators should also encourage faculty to reach out to them with concerns or questions. To facilitate this, campus units such as the faculty union or a faculty senate can function as intermediaries between faculty and administrators and can establish transparency across organizational levels, without obliging individual faculty members to publicly divulge the challenges that they are facing [16]. Additionally, interprofessional collaboration, including efforts carried out virtually, can afford an opportunity and space during COVID-19 to inspire discussions between faculty, students, staff, and administrators to collaborate to overcome challenges and consider positive lessons, solutions, and next steps [84,85].

## 6. Conclusions

At the time of this manuscript, we are experiencing increased COVID-19 positivity rates, and each new death marks a milestone in the U.S. Fortunately, we now have more scientific tools in our toolbox to slow and counter this global pandemic. What we need, now, is the desire and will to create new strategies to counteract the social determinants of health conditions (police brutality, civil unrest, structural and institutional racism, workplace discrimination) to eliminate the challenges expressed by the Black women faculty and administrators in this perspectives article. As we write this conclusion, civil unrest, protests, trauma, ACES, and forms of resistance continue throughout the U.S. It is imperative to understand, recognize, and implement the cathartic survival skills and strategies of collaboration, communication, self-care, racial and gender equity, and anti-racism through a trauma-sensitive and informed lens for students, faculty, administrators, and staff.

Through this formative perspective gathering, the authors have been able to identify future directions of inquiry and potential avenues of research that require further illumination. Future research considerations include further qualitative research into the experiences of Black women in academia; potential compilation of more experiences from BIPOC women in academia; assessing the implementation of practical tips of self-care from an individual to institutional level; and investigation of reflective and empathetic interventions conducted in varied settings such as the classroom, home, work, or school to address racial inequities in higher education. We look forward to advancing this collaborative work in the future, within which the possibilities abound.

## Figures and Tables

**Table 1:** Recommendations for institutions to support and protect Black women faculty and administrators.

Recommendation	Description/Explanation
Genuinely commit to diversity, equity, and inclusion	Have frank conversations about power, oppression, and privilege [14]. Actively recruit and retain racially diverse faculty who have diverse experiences with racism [73]. Ensure cultural competence and mentoring knowledge among faculty [76]. Provide and sustain racial awareness training for faculty [77].
Change policies to offer flexibility	Recognize the influence of gender and racial biases in student evaluations and incorporate holistic teaching assessments [16]. Adjust expectations for faculty and pause nonessential scholarship [15]. Waive nonessential services for faculty [15]. Offer more telework options. Pause the tenure clock [15].
Support professional development	Roll over unused travel funds. Sponsor professional development for faculty and staff, including certifications and professional organization memberships.
Recognize intensified caregiving demands	Acknowledge the immense health and financial tolls of the pandemic on students, faculty, and administrators. Provide emergency funds for caregiving support, including childcare and eldercare [16].
Adapt a trauma-informed approach to pedagogy	Recognize and adapt pedagogical practices that are trauma-sensitive and create resiliency during this time of unrest and chaos [78]. Understand that teaching about trauma is not the same as trauma-informed teaching [79,80].
Foster collaboration and communication	Acknowledge that outdoor community service-learning and group work are opportunities paramount to student engagement. Equip faculty to engage in positive discourse with students, lower barriers to communication, and create safe learning environments [78]. Promote interprofessional collaboration and communication [81,82]. Allow for open dialogue between faculty members and administrators [16].

## Data Availability

Not applicable.

## Key Terms in this Article

Black: Is intentionally spelled with a capital B as a form of resistance. Furthermore, Black is a form of racial and ethnic identity for people who are a part of the African diaspora.

## References

[1] Campbell T., Hribernik M. A Dangerous New Era of Civil Unrest Is Dawning in the United States and around the World. https://www.maplecroft.com/insights/analysis/a-dangerous-new-era-of-civil-unrest-is-dawning-in-the-united-states-and-around-the-world/.

[2] Xiong J., Lipsitz O., Nasri F., Lui L.M.W., Gill H., Phan L., Chen-Li D., Iacobucci M., Ho R., Majeed A. (2020). Impact of COVID-19 Pandemic on Mental Health in the General Population: A Systematic Review. J. Affect. Disord..

[3] Talevi D., Socci V., Carai M., Carnaghi G., Faleri S., Trebbi E., di Bernardo A., Capelli F., Pacitti F. (2020). Mental Health Outcomes of the COVID-19 Pandemic. Riv. Psichiatr..

[4] Galea S., Abdalla S.M. (2020). COVID-19 Pandemic, Unemployment, and Civil Unrest: Underlying Deep Racial and Socioeconomic Divides. JAMA.

[5] CDC Cases, Data, and Surveillance. https://www.cdc.gov/coronavirus/2019-ncov/covid-data/investigations-discovery/hospitalization-death-by-race-ethnicity.html.

[6] Wakeel F., Njoku A. (2021). Application of the Weathering Framework: Intersection of Racism, Stigma, and COVID-19 as a Stressful Life Event among African Americans. Healthcare.

[7] Yearby R. (2018). Racial Disparities in Health Status and Access to Healthcare: The Continuation of Inequality in the United States Due to Structural Racism: Continuing Racial Health Disparities. Am. J. Econ. Sociol..

[8] Webb Hooper M., Nápoles A.M., Pérez-Stable E.J. (2020). COVID-19 and Racial/Ethnic Disparities. JAMA.

[9] Njoku A.U. (2021). COVID-19 and Environmental Racism: Challenges and Recommendations. Eur. J. Environ. Public Health.

[10] Njoku A., Ahmed Y., Bolaji B. (2021). Police Brutality against Blacks in the United States and Ensuing Protests: Implications for Social Distancing and Black Health during COVID-19. J. Hum. Behav. Soc. Environ..

[11] Kim H., Rackoff G.N., Fitzsimmons-Craft E.E., Shin K.E., Zainal N.H., Schwob J.T., Eisenberg D., Wilfley D.E., Taylor C.B., Newman M.G. (2022). College Mental Health Before and During the COVID-19 Pandemic: Results From a Nationwide Survey. Cogn. Ther. Res..

[12] Harder W.L., McGowan B.L. Recognizing COVID-19 as Trauma: Considerations for Student Affairs Educators and Faculty Developers. https://developments.myacpa.org/recognizing-covid-19-as-trauma-considerations-for-student-affairs-educators-and-faculty-developers/.

[13] Gray K.J., Brooks L.B. (2021). Give Yourself Permission to Rest. Genealogy.

[14] Walton Q.L., Campbell R.D., Blakey J.M. (2021). Black Women and COVID-19: The Need for Targeted Mental Health Research and Practice. Qual. Soc. Work.

[15] Oleschuk M. (2020). Gender Equity Considerations for Tenure and Promotion during COVID-19. Can. Rev. Sociol./Rev. Can. Sociol..

[16] Mickey E., Clark D., Misra J. Advice to Academic Administrators for How to Best Support Faculty during the Pandemic (opinion)|Inside Higher Ed. https://www.insidehighered.com/advice/2020/09/04/advice-academic-administrators-how-best-support-faculty-during-pandemic-opinion.

[17] CDC COVID Data Tracker. https://covid.cdc.gov/covid-data-tracker.

[18] Njoku A., Joseph M., Felix R. (2021). Changing the Narrative: Structural Barriers and Racial and Ethnic Inequities in COVID-19 Vaccination. Int. J. Environ. Res. Public Health.

[19] Pew Research Center (2020). Health Concerns from COVID-19 Much Higher among Hispanics and Blacks than Whites.

[20] Sneed R.S., Key K., Bailey S., Johnson-Lawrence V. (2020). Social and Psychological Consequences of the COVID-19 Pandemic in African-American Communities: Lessons from Michigan. Psychol. Trauma.

[21] Laster Pirtle W.N., Wright T. (2021). Structural Gendered Racism Revealed in Pandemic Times: Intersectional Approaches to Understanding Race and Gender Health Inequities in COVID-19. Gender Soc..

[22] Alang S., McAlpine D., McCreedy E., Hardeman R. (2017). Police Brutality and Black Health: Setting the Agenda for Public Health Scholars. Am. J. Public Health.

[23] Jee-Lyn García J., Sharif M.Z. (2015). Black Lives Matter: A Commentary on Racism and Public Health. Am. J. Public Health.

[24] De Gue S., Fowler K.A., Calkins C. (2016). Deaths Due to Use of Lethal Force by Law Enforcement. Am. J. Prev. Med..

[25] Bolin B., Kurtz L.C., Rodríguez H., Donner W., Trainor J.E. (2018). Race, Class, Ethnicity, and Disaster Vulnerability. Handbook of Disaster Research.

[26] Fothergill A., Maestas E.G.M., Darlington J.D. (1999). Race, Ethnicity and Disasters in the United States: A Review of the Literature. Disasters.

[27] Feldman J.M., Chen J.T., Waterman P.D., Krieger N. (2016). Temporal Trends and Racial/Ethnic Inequalities for Legal Intervention Injuries Treated in Emergency Departments: US Men and Women Age 15–34, 2001–2014. J. Urban Health.

[28] Krieger N., Chen J.T., Waterman P.D., Kiang M.V., Feldman J. (2015). Police Killings and Police Deaths Are Public Health Data and Can Be Counted. PLoS Med..

[29] Rouhandeh A.J. Social Distancing, Racism, and Protecting People in a Pandemic without the Police. https://prospect.org/api/content/58267086-b11f-11ea-86d9-1244d5f7c7c6/.

[30] Onwuamaegbu N. Many Black Women Felt Relieved to Work from Home, Free from Microaggressions. Now They’re Told to Come Back. https://www.washingtonpost.com/lifestyle/2021/07/24/black-women-office-work-home/.

[31] Lloyd C. Black Women in the Workplace. https://www.gallup.com/workplace/333194/black-women-workplace.aspx.

[32] Landertinger L.C.L., Hopson A., Cooper M. Emotional and Mental Health Support for Black Students: Responding to Racial Trauma and White Terror amidst COVID 19 Laura C. L. Landertinger, Anita Hopson, Elijah Greene, and Miracle Cooper. https://issuu.com/aaua10/docs/twin_pandemics/s/11997062.

[33] Bor J., Venkataramani A.S., Williams D.R., Tsai A.C. (2018). Police Killings and Their Spillover Effects on the Mental Health of Black Americans: A Population-Based, Quasi-Experimental Study. Lancet.

[34] Hawkins D.S. (2021). “After Philando, I Had to Take a Sick Day to Recover”: Psychological Distress, Trauma and Police Brutality in the Black Community. Health Commun..

[35] Malone Gonzalez S. (2019). Making It Home: An Intersectional Analysis of the Police Talk. Gender Soc..

[36] Joe J.R., Shillingford-Butler M.A., Oh S. (2019). The Experiences of African American Mothers Raising Sons in the Context of #BlackLivesMatter. TPC.

[37] Jackson F.M., James S.A., Owens T.C., Bryan A.F. (2017). Anticipated Negative Police-Youth Encounters and Depressive Symptoms among Pregnant African American Women: A Brief Report. J. Urban Health.

[38] Dreyer B.P. (2021). The Toll of Racism on African American Mothers and Their Infants. JAMA Netw. Open.

[39] Young J.L., Hines D.E. (2018). Killing My Spirit, Renewing My Soul: Black Female Professors’ Critical Reflections on Spirit Killings While Teaching. Women Gender Fam. Color.

[40] Barry D., McIntire M., Rosenberg M. ‘Our President Wants Us Here’: The Mob That Stormed the Capitol. https://www.nytimes.com/2021/01/09/us/capitol-rioters.html.

[41] Morrison A. Race Double Standard Clear in Rioters’ Capitol Insurrection. https://apnews.com/article/congress-storming-black-lives-matter-22983dc91d16bf949efbb60cdda4495d.

[42] Whitford E. A Day Later, College Presidents Continued to Condemn Violence at the Capitol. https://www.insidehighered.com/news/2021/01/08/day-later-college-presidents-continued-condemn-violence-capitol.

[43] Grullón Paz I. Pandemic and Racial Injustice Cause Outsize Harm to Black Students, Study Finds. https://www.nytimes.com/2021/07/27/us/covid-race-impact-black-education.html.

[44] Butler J.E. Teaching after the Insurrection: Belonging and Othering in the Classroom. https://www.aacu.org/liberaleducation/2021/summer/butler.

[45] Horsford S.D., Cabral L., Touloukian C., Parks S., Smith P.A., McGhee C., Qadir F., Lester D., Jacobs J. (2021). Black Education in the Wake of COVID-19 & Systemic Racism.

[46] Gould E., Wilson V. (2020). Black Workers Face Two of the Most Lethal Preexisting Conditions for Coronavirus—Racism and Economic Inequality.

[47] Chance N.L. (2021). A Phenomenological Inquiry into the Influence of Crucible Experiences on the Leadership Development of Black Women in Higher Education Senior Leadership. Educ. Manag. Adm. Leadersh..

[48] The Condition of Education 2020 (NCES 2020-144), Characteristics of Postsecondary Faculty. https://nces.ed.gov/programs/coe/indicator/csc#fn2.

[49] Harris A. The Disciplines Where No Black People Earn Ph.D.s. https://www.theatlantic.com/education/archive/2019/04/lack-of-black-doctoral-students/587413/.

[50] De Brey C., Musu L., McFarland J., Wilkinson-Flicker S., Diliberti M., Zhang A., Branstetter C., Wang X. Status and Trends in the Education of Racial and Ethnic Groups 2018. NCES 2019-038. https://eric.ed.gov/?id=ED592833.

[51] American College President Study (ACPS). https://www.acenet.edu/Research-Insights/Pages/American-College-President-Study.aspx.

[52] Walkington L. (2017). How Far Have We Really Come? Black Women Caculty and Graduate Students’ Experiences in Higher Education. Humboldt J. Soc. Relat..

[53] Spraggins R.E. (1998). Fly Girl in the Buttermilk: The Graduate Experiences at a Predominantly White Institution of Black Women Who Attended Various Undergraduate Environments. Ph.D. Dissertation.

[54] Wilson S., Muhs G.G. (2012). They Forgot Mammy Had a Brain. Presumed Incompetent: The Intersections of Race and Class for Women in Academia.

[55] Aparicio F.R. (1999). Through My Lens: A Video Project about Women of Color Faculty at the University of Michigan. Fem. Stud..

[56] Turner C.S.V. (2002). Women of Color in Academe: Living with Multiple Marginality. J. High. Educ..

[57] Taylor E., Gillborn D., Ladson-Billings G. (2009). Foundations of Critical Race Theory in Education.

[58] Lechuga-Peña S. (2022). Navigating Pre-Tenure and COVID-19: A Testimonio of a BIPOC Junior Faculty Mother. Affilia.

[59] Johnson T.A., Bankhead T. (2014). Hair It Is: Examining the Experiences of Black Women with Natural Hair. JSS.

[60] Crenshaw K. (1991). Mapping the Margins: Intersectionality, Identity Politics, and Violence against Women of Color. Stanf. Law Rev..

[61] Erete S., Rankin Y.A., Thomas J.O. (2021). I Can’t Breathe: Reflections from Black Women in CSCW and HCI. Proc. ACM Hum.-Comput. Interact..

[62] Davis D.R., Maldonado C. (2017). Shattering the Glass Ceiling: The Leadership Development of African American Women in Higher Education. Adv. Women Leadersh. J..

[63] Washington N., Grady S.D., McMullen K., Daily S., Marshall B. Flowing, Not Forcing: Finding and Maintaining Authenticity as Black Women in Academia. Proceedings of the 52nd ACM Technical Symposium on Computer Science Education.

[64] King M.M., Frederickson M.E. (2021). The Pandemic Penalty: The Gendered Effects of COVID-19 on Scientific Productivity. Socius.

[65] Cui R., Ding H., Zhu F. (2020). Gender Inequality in Research Productivity during the COVID-19 Pandemic. SSRN J..

[66] Harris A. The Death of an Adjunct. https://www.theatlantic.com/education/archive/2019/04/adjunct-professors-higher-education-thea-hunter/586168/.

[67] Flaherty C. Study Finds Gains in Faculty Diversity, but Not on the Tenure Track. https://www.insidehighered.com/news/2016/08/22/study-finds-gains-faculty-diversity-not-tenure-track.

[68] Aviles G. The Coronavirus Is Threatening Diversity in Academia. https://www.nbcnews.com/news/us-news/coronavirus-threatening-diversity-academia-n1212931.

[69] Mosley M.H. (1980). Black Women Administrators in Higher Education: An Endangered Species. J. Black Stud..

[70] Wolfe B.L. (2010). When Being Black Isn’t Enough: Experiences and Persistence Strategies of Six African American Administrators at a PWI. ProQuest Dissertations. Ph.D. Dissertation.

[71] Pointer R.A. (2021). African American Administrators’ Perceptions of Persistence and Retention Obstacles for African American Students Enrolled at Predominately White Universities. ProQuest Dissertations & Theses Global. Ph.D. Dissertation.

[72] Gardner L., Barrett T.G., Pearson L.C. (2014). African American Administrators at PWIs: Enablers of and Barriers to Career Success. J. Divers. High. Educ..

[73] Hoyt L.T., Cohen A.K., Dull B., Maker Castro E., Yazdani N. (2021). “Constant Stress Has Become the New Normal”: Stress and Anxiety Inequalities among U.S. College Students in the Time of COVID-19. J. Adolesc. Health.

[74] Ramey F.H. (1995). Obstacles Faced by African American Women Administrators in Higher Education: How They Cope. West. J. Black Stud..

[75] Nicol D.J., Yee J.D. (2017). “Reclaiming Our Time”: Women of Color Faculty and Radical Self-Care in the Academy. Fem. Teach..

[76] Wilson A.H., Sanner S., McAllister L.E. (2010). A Longitudinal Study of Cultural Competence among Health Science Faculty. J. Cult. Divers..

[77] Diggles K. (2014). Addressing Racial Awareness and Color-Blindness in Higher Education: Addressing Racial Awareness and Color-Blindness in Higher Education. New Dir. Teach. Learn..

[78] Friedrich S., Straub C., Bode S.F.N., Heinzmann A. (2021). SIESTA: A Quick Interprofessional Learning Activity Fostering Collaboration and Communication between Paediatric Nursing Trainees and Medical Students. BMC Med. Educ..

[79] Wynard T., Benes S., Lorson K. (2020). Trauma-Sensitive Practices in Health Education. J. Phys. Educ. Recreat. Dance.

[80] CDC Preventing Adverse Childhood Experiences. https://www.cdc.gov/vitalsigns/aces/index.html.

[81] Carello J., Butler L.D. (2014). Potentially Perilous Pedagogies: Teaching Trauma Is Not the Same as Trauma-Informed Teaching. J. Trauma Dissociation.

[82] Crosby S.D. (2015). An Ecological Perspective on Emerging Trauma-Informed Teaching Practices. Child. Sch..

[83] Dias I.F., Bellucci C. (2021). Class Representatives and Students’ Behaviour: An Overview at the Post-COVID-19 Pandemic in the Perspective of the Community of Practice. Int. J. Educ. Stud..

[84] Lackie K., Najjar G., El-Awaisi A., Frost J., Green C., Langlois S., Lising D., Pfeifle A.L., Ward H., Xyrichis A. (2020). Interprofessional Education and Collaborative Practice Research during the COVID-19 Pandemic: Considerations to Advance the Field. J. Interprof. Care.

[85] Khalili H. (2020). Online Interprofessional Education during and Post the COVID-19 Pandemic: A Commentary. J. Interprof. Care.

